# How Neoliberal are You? Development and Validation of the Neoliberal Orientation Questionnaire

**DOI:** 10.5334/irsp.663

**Published:** 2023-07-19

**Authors:** Lola Girerd, John T. Jost, Virginie Bonnot

**Affiliations:** 1 Laboratoire de Psychologie Sociale, Université Paris Cité, FR; 2 Department of Psychology, New York University, US

**Keywords:** Neoliberal ideology, NOQ, scale development, validation, economic system justification

## Abstract

We created a novel instrument to assess individual orientations toward the neoliberal capitalist system, the Neoliberal Orientation Questionnaire (NOQ), which is comprised of four dimensions: competitiveness, individual self-regulation, relational detachment, and public divestment. The instrument was intended to complement existing scales by (a) adopting a European perspective, and (b) incorporating personal as well as societal values, including lifestyle considerations. We sought to validate the NOQ in a European country with a strong history of public investment and social welfare provisions, namely France. In three nationally representative French samples, and one US student sample we assessed the internal consistency and construct validity of long and short versions of the scale. In terms of convergent and divergent validity, NOQ scores were positively correlated with scores on the Neoliberal Beliefs Inventory (NBI), general and economic forms of system justification, social dominance orientation, social and economic conservatism, internal locus of control, belief in free will, future-orientation, and a tendency to look on the ‘bright side’ in the face of hardships. The NOQ should prove useful for understanding the antecedents, concomitants, and consequences of attitudinal support versus opposition to the neoliberal capitalist system that dominates contemporary Western societies.

‘The ideology that dominates our lives has, for most of us, no name.’

(George Monbiot, 2016)

Neoliberal ideology may be understood both as a means of legitimizing political and economic practices under contemporary free market capitalism and a way in which individuals adapt themselves to such practices (e.g., Azevedo et al., 2019; Binkley, 2011a; Harvey, 2007; Monbiot, 2016). As such, neoliberal ideology involves an element of *injunctification*, that is, using beliefs, opinions, and values to convert what *is*—a free market economy that is privatizing many formerly public institutions, organizations, and services—into what *should* be (Kay et al., 2009). In this sense, neoliberal ideology is system-justifying, insofar as it leads people to become more comfortable and satisfied with the political and economic status quo and less likely to question or challenge it (Jost, 2020).

For example, one major focus of neoliberal policies involves public divestment, that is, a retrenchment of the State from its formerly supportive and regulatory roles in social and economic affairs, enabling private, profit-oriented companies to fill the void (Harvey, 2007). The ideology that helps to rationalize such a retrenchment places a heavy discursive emphasis on themes of personal responsibility (e.g., ‘It’s not up to the State to “take care” of its citizens, it’s up to individuals themselves’; see Hache, 2007; Pyysiäinen, Halpin & Guilfoyle, 2017). In this way, the ideology also encourages citizens to take it upon themselves to increase their own human capital—that is, personal attributes and skills they can invest in for the sake of future returns (Becker, 1993)—to succeed in a capitalist system.

To justify the highly competitive social and economic practices that are integral to contemporary capitalism, neoliberal ideology not only valorizes personal responsibility and individual effort but also the necessity of competition and the benefits it ostensibly brings (e.g., Bay-Cheng et al., 2015; Girerd, Verniers & Bonnot, 2021; Pulfrey & Butera, 2013). To legitimize the economic rewards that flow disproportionately to the ‘winners’ of economic competition, a meritocratic ideology is required, so that inequality is justified (or, more precisely, justicized) in terms of ability, talent, effort, motivation, and other indicators of personal deservingness (e.g., Bettache, Chiu & Beattie, 2020; Darnon, Smeding & Redersdorff, 2017; Frank, 2016; Jost & Kay, 2010). The deregulation of labor practices and the demise of employee unions under contemporary capitalism is often defended and justified by the rhetoric of individual freedom and flexibility (e.g., Eagleton-Pierce, 2016; Harvey, 2007). Finally, by promoting the ideal of the self-employed worker, as exemplified by the Uber model, large companies are freed from the burden of having legal and other obligations to their employees, who must adapt to the new economy or risk becoming disposable.

On this conception, neoliberal ideology involves several discursive and motivational elements (Jost, Federico & Napier, 2009), some of which are explicitly political, while others are seemingly personal or private (e.g., Girerd et al., 2020, Study 2). Over time, citizens not only come to accept the ideological rationale for public divestment and the dismantling of social welfare programs, they develop a kind of neoliberal form of rationality in which cost-benefit analyses are applied to all areas of their lives, including personal relationships and educational and career choices (Arfken, 2018; McDonald & O’Callaghan, 2008; Ratner, 2019; Teo, 2018). A neoliberal rationality is one that, in addition to upholding principles of personal responsibility and deservingness, also focuses on individual happiness and the quest for positive emotion in general (Adams et al., 2019; Binkley, 2011b; Hache, 2007). Like other system-justifying belief systems, neoliberal ideology may serve the palliative function of making people more contented with their lot in life, for better or worse (Girerd, Verniers & Bonnot, 2021; Jost, 2020).

The neoliberal economic model, as an extension and intensification of free-market capitalism (Ratner, 2019), has now spread to most countries in the world, especially in the West (Harvey, 2007; Navarro, 2007). At the same time, it must adapt itself to specific country-level contexts, and so must the ideology that accompanies and justifies it (Arfken, 2018). For instance, in France, where the present research program was mainly conducted, people are often not strong defenders of public divestment. Instead, French citizens still expect the State to ensure a certain level of public service and to guarantee equality of opportunity (Girerd, Verniers & Bonnot, 2021). France is, in fact, an especially interesting context in which to study neoliberal ideology. This is because neoliberal rhetoric and policy, which began in the 1970s and has been increasing ever since (Dardot & Laval, 2019; Foucault, 2004a), has coexisted alongside the persistent egalitarian ideals—and concerns about poverty—that are reflected in French attachment to the welfare state (Jetten, Mols & Selvanathan, 2020; Krauth-Gruber & Bonnot, 2020; Langer et al., 2020; Le Figaro, 2018). We propose that neoliberal ideology possesses core features that are pertinent to many countries, as well as other features that may be specific to certain sociopolitical contexts. In the present research program, our goal was not to develop a scale that would only capture aspects that were idiosyncratic to France. Rather, we aimed to develop a scale that would be well-suited but not necessarily limited to the French context. For example, we did not emphasize the desire for State retrenchment as much as we would have if we had focused primarily on the United States (Girerd, Verniers & Bonnot, 2021).

If researchers wish to investigate how neoliberal systems affect individuals, and by extension social groups or even entire communities, they must have at their disposal appropriate tools or instruments that are capable of capturing such a complex and multi-faceted concept as neoliberal ideology. To our knowledge, there are two scales that have been used to measure acceptance versus rejection of neoliberal ideology: the Neoliberal Beliefs Inventory (NBI; Bay-Cheng et al., 2015) and the Anti-Neoliberal Attitudes Scale (ANAS; Grzanka et al., 2020). We believe that these questionnaires are useful and capture some very important aspects of neoliberal ideology. At the same time, they are subject to certain limitations that we seek to overcome by introducing an additional, complementary instrument.

First, as readily acknowledged by the original authors, the NBI and ANAS were developed and validated in the context of the US. For this reason, these scales mention affirmative action and other issues that are not relevant to many other countries. Second, the NBI is comprised of four dimensions—system inequality, competition, personal wherewithal, and government interference—that focus more or less exclusively on political and economic attitudes. While we regard these as very important, we also believe that there are important personal or private aspects of neoliberal ideology and the psychological mindset that accompanies it (e.g., Binkley, 2011b; Foucault, 2004b; Pyysiäinen et al., 2017). Third, items on the government interference dimension of the NBI, such as ‘The government does not have a right to take what I earn and give it to someone else,’ may be rejected for reasons having little or nothing to do with opposition to neoliberalism in a country like France with a very strong tradition of Statism and egalitarianism (Girerd, Verniers & Bonnot, 2021). Fourth, the ANAS is comprised of four value dimensions, namely racism and sexism awareness, communitarianism, multiculturalism, and inequality consciousness, that may be closely tied to the ideologies of liberalism and conservatism (Jost, 2021). Indeed, the ANAS combines items from scales that were not originally designed to measure neoliberal ideology, such as the Color-blind Racial Attitudes Scale. Moreover, the scale measures *anti*-neoliberal attitudes rather than neoliberal attitudes per se. Like the NBI, the ANAS fails to capture more personal or private aspects of neoliberal ideology, which we sought to include in the NOQ.

Finally, both scales focus a great deal on (lack of) perception of inequalities, and as such imply that it invariably characterizes neoliberal ideology. However, the existence of neoliberal feminism (Rottenberg, 2018) suggests that it is possible for people to acknowledge and oppose at least some degree of gender inequality while still embracing a neoliberal outlook (Fitz et al., 2012). For this reason, we put more conceptual space between attitudes about inequality, on one hand, and neoliberal ideology, on the other, in comparison with both the NBI and the ANAS.

In summary, then, we sought to develop a multifactorial scale of neoliberal ideology that would (1) be deeply informed by qualitative interview data, (2) apply to private as well as public domains, (3) enable us to consider perceptions of inequality as a possible consequence of neoliberal ideology rather than a constituent element, and (4) have psychological resonance in Europe as well as other regions, such as North America, in which neoliberal conceptions of free market capitalism have spread.

With all of this in mind, we created a tool for assessing the individual’s ideological orientation toward neoliberalism (i.e., the Neoliberal Orientation Questionnaire; NOQ). In developing the items, we drew heavily upon qualitative data acquired through semi-structured narrative interviews conducted in France (Girerd, Verniers & Bonnot, 2021), which, as noted above, is a country with a long history of Statism, as well as a more recent interest in neoliberal economic models. In the first three studies presented here, we sought to validate the NOQ in nationally representative samples of men and women in France. This is an important advance, insofar as the NBI and ANAS were validated using (mostly) student samples. Despite the initial focus on France, we hoped that the measure would prove useful in other contexts as well. Thus, in a fourth study we administered a shorter version of the scale (NOQ-S) to a sample of university students in the US to further probe convergent validity.^1^ For this short version, we selected only those items that seemed to represent each dimension well enough and also to apply well to the US context.

## Hypotheses

In terms of theoretical advancement, we conceive of neoliberal ideology, as noted above, as a system-justifying ideology that lends legitimacy to contemporary instantiations (and idealizations) of laissez-faire capitalism (Jost, 2020). This economic model is notable for its public divestment from the policies and institutions of social welfare liberalism that characterized many Western societies in the aftermath of the Second World War, as well as its ideological emphasis on the importance of personal responsibility, individual effort and self-regulation, competitiveness in business and other aspects of life, meritocratic principles of resource distribution, the pursuit of happiness, and the entrepreneurial spirit. Thus, we would hypothesize that endorsement of neoliberal ideology would be positively associated with both general and economic forms of system justification, that is, the tendency to regard the societal status quo and the capitalist economic system in particular as fair, natural, legitimate, and desirable (Azevedo et al., 2019; Girerd & Bonnot, 2020; Girerd et al., 2020; Jost, 2020).

It has been suggested that neoliberal ideology—especially support for laissez-faire capitalism—is best characterized as libertarian, that is, an orientation that is economically conservative but socially liberal (e.g., Feldman & Johnston, 2014; Iyer et al., 2012). From this perspective, one would predict that scores on the NOQ would be positively associated with economic conservatism but negatively associated with social or cultural conservatism. By contrast, other researchers have proposed that ideological defense of the capitalist system involves a justification not only of economic disparities, but also of social disparities—such as those arising from racial, ethnic, gender, and social class distinctions—that are legitimized by deflecting blame from the social system and placing it squarely on those individuals who are deemed ‘incapable’ of climbing the economic ladder (e.g., Azevedo et al., 2019; Giroux, 2004; Monbiot, 2016; Sidanius & Pratto, 1993). From this latter, more critical perspective, one would predict that scores on the NOQ would be positively associated not only with economic system justification but also social conservatism, right-wing orientation, and social dominance orientation, operationalized as a general (i.e., not purely economic) preference for group-based hierarchy in society.

With regard to gender issues, it has been proposed that neoliberal ideology is likely to impede the process of feminist identification and support for collective action on behalf of the group of women (Girerd & Bonnot, 2020). This is because the feminist label implies a politicized identity involving the recognition that women are a disadvantaged group relative to men, that the state of disadvantage is structurally determined and reproduced, and that collective—rather than purely personal—action is required (Gurin, Miller & Gurin, 1980; Simon & Klandermans, 2001; van Stekelenburg & Klandermans, 2013; Zucker & Bay-Cheng, 2010). Neoliberal ideology, on the other hand, assumes that people are to blame for their own problems, and that they should seek to find individual solutions to their problems, mainly by transforming themselves (Binkley, 2011a; Girerd, Verniers & Bonnot, 2021; Kim, Fitzsimons & Kay, 2018).

Because having a politicized identity is a very strong predictor of participation in collective action (van Zomeren, Postmes & Spears, 2008) and neoliberal ideology is likely to discourage people from engaging in collective action (Girerd & Bonnot, 2020; Zucker & Bay-Cheng, 2010), we hypothesized that NOQ scores would be negatively associated with (a) feminist identification and (b) engagement in collective action (as has already been demonstrated with respect to the NBI; Bay-Cheng et al., 2015). Moreover, to the extent that neoliberal ideology abstracts people from their material and social contexts (Adams et al., 2019; Girerd, Verniers & Bonnot, 2021), and focuses instead on individual self-regulation, it should lead people to disregard the influence that their groups’ memberships have on their lives—in the form of privilege for advantaged group members and a lack of privilege for disadvantaged group members. For these reasons, we also expect a negative correlation between the NOQ-S and perceived gender discrimination for women.

On the other hand, as mentioned above, some have argued that it is indeed possible to be a ‘neoliberal feminist’ (Bongiorno et al., 2021; Rottenberg, 2018), that is, a woman who holds certain egalitarian goals about gender, as long as they do not conflict with meritocratic principles, but also seeks personal advancement and professional success in the context of the highly competitive free-market system (Fitz, Zucker & Bay-Cheng, 2012; Rottenberg, 2018). Thus, an important goal of the present research program was to understand the nature of the relationship between neoliberal ideology and attitudes about gender among women. Because the NOQ does not include items that are focused explicitly on attitudes about social equality, as noted above, we were able to investigate the extent to which a neoliberal orientation is or is not incompatible with the holding of feminist identity and perceptions of gender discrimination.

We also sought to illuminate the more personal—and less overtly political—aspects of the neoliberal orientation. In light of the foregoing analysis, we hypothesized that scores on the NOQ would be positively associated with belief in free will (Caspar et al., 2017); personal growth initiative (Robitschek et al., 2012); and a preference for internal (vs. external) explanations for human behavior (Dubois & Beauvois, 2005; Kim, Fitzsimons & Kay, 2018; Valecha & Ostrom, 1974). Likewise, because neoliberal ideology promotes a quest for personal happiness and encourages people to be both resilient and adaptive (Binkley, 2011b), we hypothesized that NOQ scores would be positively related to the tendency to find ‘silver linings’ in the context of personal hardships. Moreover, in inviting people to shape and invest in their human capital (Arfken, 2018), neoliberal ideology requires future-oriented choices and behaviors, working in the present for future returns (De La Fabián & Stecher, 2017). Therefore, we also expected a positive correlation between NOQ scores and taking into account the future consequences of one’s present actions.

To assess incremental validity, we administered the NBI scale (Bay-Cheng et al., 2015). Because of concerns about survey length, we were unable to administer both the NBI and the ANAS. We chose to administer the NBI rather than the ANAS because (a) the former has already been used in research in France (Girerd & Bonnot, 2020), and (b) it is closer to the NOQ in terms of substantive content, as described above. We also included a measure of social desirability to ensure that NOQ scores were not unduly driven by such concerns.

## Method

### Item Development

Relying on a mixed method approach (Creswell, 2014), item development was based on the qualitative analysis of 32 semi-structured interviews that, along with a literature review, revealed five dimensions of neoliberal ideology in the French context (Girerd, Verniers & Bonnot, 2021): State prerogatives (the extent to which the government should or should not intervene in social and economic affairs), competition (the extent to which competition is perceived as something necessary and desirable), abstraction from structural, normative, and social contexts (the perception that outside influences are detrimental and should be avoided), the entrepreneurial self (aiming for personal growth, self-regulation, and self-mastery), and emotional management (striving for personal happiness through emotion regulation).

Based on these categories an initial pool of 136 items was generated. In constructing these items, we sought to capture the precise language used by interview participants. We also sought to include items that would be highly pertinent but not restricted to the context of France. Three pilot studies (total *N* = 778) followed by deliberations involving the first and third authors led to the removal of ambiguous and redundant items and those that exhibited floor or ceiling effects, as well as linguistic refinement of the remaining items, and the addition of several items based on the literature review. This process yielded a pool of 68 items.

### Participants and Procedure

A French polling company (IPSOS) was hired to recruit three independent samples that approximated national representativeness based on sample sizes of 600 (Sample 1), 500 (Sample 2) and 250 (Sample 3). The sample sizes of the three studies were decided at the same time because we hired IPSOS to conduct the whole set of studies, and the cost was based on the total number of participants.

French participants were compensated by IPSOS with points they could use as gift cards. The number of points was dependent upon the duration of the study. In the three French samples, the age range was limited to 18–50 because we wanted participants who had lived under neoliberalism for most or all of their lives. Quotas were set on age groups, French regions, and gender (except for Sample 3, which included only women).

In a fourth study we administered a 12-item version of the scale (NOQ-S, with 3 items per dimension) to 452 introductory psychology students in the United States, who completed a mass-testing battery to satisfy a course requirement. This enabled us to explore additional hypotheses in a different cultural context.

In the first three samples, we excluded respondents who were not French nationals (*n* = 20 in Sample 1, *n* = 13 in Sample 2, *n* = 9 in Sample 3), those who failed to complete all of the scales (*n* = 35 in Sample 1, *n* = 37 in Sample 2, *n* = 12 in Sample 3), who gave bogus answers (e.g., the same score for all items; *n* = 16 in Sample 1, *n* = 11 in Sample 2, *n* = 1 in Sample 3) and those who completed the study in less than one-third of the mean average response time (*n* = 24 in Sample 1, *n* = 3 in Sample 2, *n* = 21 in Sample 3). Although we neglected to include an attention check in Sample 1, we did use one in Samples 2 and 3 to screen out participants who failed to read the instructions properly. In Sample 4, we excluded participants who failed to complete the NOQ-S in its entirety (*n* = 3). Thus, the final sample sizes were 580, 476, 235 and 449 participants, respectively.

For Sample 1 we hoped to rely on the 10 participants per item recommendation for factor analysis (Costello & Osborne, 2005). However, we set the limit to 600 participants because of budget limitations. For Samples 2 and 3 we tried to obtain the largest possible samples given our budget limitations. Fortunately, Sample 2 exceeds the requirements for similar CFAs (e.g., about 200 participants are recommended for models of 3 factors including about 6 items per factor with factor loadings of .50; Wolf et al., 2013). A sensitivity analysis revealed that Sample 3 was large enough to detect small to medium effect sizes equivalent to *n²* = 0.04 with 80% power and an alpha level of .05, for a multiple regression analysis with three predictors. Finally, Sample 4 was large enough to obtain stable correlation estimates (Schönbrodt & Perugini, 2013). Moreover, for an alpha level of .05 and with 80% power, sensitivity analyses revealed that Samples 1, 2, 3 and 4 were large enough to reliably detect correlations ranging from |.12| to |.18| in the population.

The purpose of Study 1 was to perform an Exploratory Factor Analysis and to identify and eliminate items with poor psychometric properties. The purpose of Study 2 was to perform a Confirmatory Factor Analysis. The purpose of Study 3 was to provide additional information concerning incremental validity of the NOQ in relation to the NBI. In Study 4, we explored the reliability of the NOQ-S in the US context. In addition, all studies were used to assess the internal consistency and sensitivity of the NOQ (and its subscales) and to provide information concerning its validity.

All participants provided informed consent before answering the questionnaires. In Studies 1–3 we included measures of participants’ sex, age, nationality, socio-professional group, subjective socio-economic status, political orientation, and perceived proximity to a French political party (see Supplementary Material 1 for detailed information). In Study 4, we assessed participants’ age, gender identification, sex assigned at birth, year of enrollment in university, level of English fluency, and political orientation. Socio-demographic information pertaining to the final samples is provided in Table 1.

**Table 1:** Socio-Demographic Information Pertaining to Samples 1–4.


SOCIO-PROFESSIONAL GROUP												

	AGE *M* (SD)	FEMALE	SES	POL ORIENT	FARMERS	CRAFTMEN/WOMEN, STOREKEEPERS, BUSINESS OWNERS	MANAGERS, HIGHER INTELLECTUAL PROFESSIONS	INTERMEDIATE PROFESSIONS	EMPLOYEES	FACTORY WORKERS	RETIRED	OTHER WITH NO OCCUPATIONAL ACTIVITY

**Sample 1** (*N* = 580) FR	34.79 (9.45)	48.97%	5.62 (1.67)	5.08 (1.85)	3	27	109	112	196	56	1	76

**Sample 2** (*N* = 476) FR	34.89 (9.38)	49.79%	5.32 (1.85)	5.00 (1.78)	2	15	94	65	150	21	2	127

**Sample 3** (*N* = 235) FR	34.50 (9.48)	100%	5.32 (1.61)	4.97 (1.59)	1	6	30	38	106	3	0	51

	**Age** *M* (SD)	**Female** (gender identity)	**Female** (sex at birth)	**Pol Orient**	**Year of enrollment** (1–4)				**English fluency** (1 = not at all fluent; 5 = completely fluent)			

**Sample 4** (*N* = 449) US	19.42 (1.45)	68.75%	71.05%	4.26 (1.91)	1^st^ = 158; 2^nd^ = 183; 3^rd^ = 64; 4^th^ = 44				1 = 0; 2 = 7; 3 = 47; 4 = 64; 5 = 331			


*Note*: SES = Subjective Socio-Economic Status ranging from 1 to 10, higher scores representing a higher subjective SES. Pol Orient = Political Orientation ranging from 1 (extreme left) to 9 (extreme right) for Samples 1–3, and from 1 (extremely liberal) to 11 (extremely conservative) for Sample 4.

### Measures

#### Convergent Validity

For measures used to establish convergent validity, responses were provided on 7-point scales ranging from ‘totally disagree’ to ‘totally agree,’ unless otherwise specified.

*Economic System Justification (Samples 1 & 4)*. We administered the 17-item Economic System Justification scale (Sample 1, ω = 0.81; Sample 4, ω = 0.86; Jost & Thompson, 2000) assessing participants’ legitimation of economic inequality under capitalism (e.g., ‘There will always be poor people, because there will never be enough jobs for everybody’).

*Free Will (Sample 1)*. We administered Caspar et al.’s (2017) 7-item scale (ω = 0.78) assessing participants’ belief in free will (e.g., ‘People have complete control over the decisions they make’).

*Personal Growth Initiative (Sample 1)*. We administered Robitschek et al.’s (2012) 16-item Personal Growth Initiative scale (ω = 0.91) which is comprised of 4 dimensions (planfulness, readiness for change, using resources, and intentional behavior) and that measures personal involvement in one’s own development (e.g., ‘I take every opportunity to grow as it comes up’).

*General System Justification (Samples 2 & 4)*. The 8-item General System Justification scale was used to assess participants’ evaluation of the fairness of French (Sample 2) and US (Sample 4) societies overall (Sample 2, ω = 0.84; Sample 4, ω = 0.84; Kay & Jost, 2003; e.g., ‘In general, the French political system works as it should’).

*Social conservatism (Samples 2 & 4)*. In Sample 2, we administered the 7-item social conservatism subscale (ω = 0.78) from Everett’s (2013) Social and Economic Conservatism Scale, which requires participants to rate how positively or negatively they feel about 7 sociopolitical themes (e.g., ‘traditional values’) on a scale ranging from 0 (‘very negative’) to 100 (‘very positive’). In Sample 4, we administered a single item: ‘In terms of social and cultural issues, how liberal or conservative are you?’ ranging from 1 ‘extremely liberal’ to 11 ‘extremely conservative.’

*Locus of Control (Sample 2)*. Participants’ preferences for internal vs. external causal explanations were measured with the 11-item Locus of Control scale (Valecha & Ostrom, 1974). One item was removed to increase internal consistency (ω = 0.64). Participants were asked to indicate which of two statements was closest to their view. In each case, one statement captured an internal and the other an external explanation (e.g., ‘Many of the unhappy things in people’s lives are partly due to bad luck’ vs. ‘People’s misfortunes result from the mistakes they make’). Participants were also asked to indicate how close the chosen statement was to their own view by selecting either ‘slightly closer’ or ‘much closer.’ The total score corresponds to the sum of values and can therefore range from 11 to 44. Responses were coded so that a higher score would indicate a stronger preference for internal over external causal explanations.

*Silver Lining (Sample 2)*. We relied on the 24-item Silver Lining Questionnaire to assess participants’ ability to find positive elements in difficult experiences (ω = 0.93; Bride et al., 2008). Participants were asked to identify one difficult event that had occurred in their lives and to answer the questions with this event in mind. Although the original scale focused on coping with disease, we adapted the scale so that participants could describe any difficult life event (e.g., ‘This hardship gave me more confidence’).^2^

*Feminist identification (Sample 3)*. We administered the feminist identification scale used by Girerd and Bonnot (2020, based on Luhtanen & Crocker, 1992, and Szymanski, 2004) but removed two items that focused on the goals of feminism, because we were interested in assessing feminist identification rather than perceptions of the movement’s goals. This produced a 5-item scale (ω = 0.88; e.g., ‘I consider myself a feminist’).

*Collective action (Sample 3)*. Participants’ self-reported engagement in collective action on behalf of women was assessed with Foster and Matheson’s (1995) 25-item Collective Action Scale (e.g., ‘I have participated in protests regarding women’s issues’). Participants were asked to indicate whether they had engaged in any of the 25 behaviors in the past 6 months using a ‘Yes/No’ format. Responses were summed to estimate the total number of behaviors, so that scores could range from 0 to 25.

*Social Dominance Orientation (Sample 3)*. We administered the 16-item Social Dominance Orientation scale that was validated in France (ω = 0.91; Duarte, Dambrun & Guimond, 2004, based on Pratto et al., 1994). It contains two subscales, group-based dominance (e.g., ‘Certain groups of people are simply inferior to other groups’) and opposition to equality (e.g., ‘Group equality should be our ideal,’ reverse-coded; see Jost & Thompson, 2000).

*Economic conservatism (Sample 4)*. We administered one item: ‘In terms of economic issues, how liberal or conservative are you?’ ranging from 1 ‘extremely liberal’ to 11 ‘extremely conservative.’

*Orientation toward the future (Sample 4)*. We used the Consideration of Future Consequences (CFC-14) scale to assess the extent to which participants are future-oriented when they consider their present behavior (Joireman et al., 2012; ω = 0.86; e.g., ‘My behavior is generally influenced by future consequences’), ranging from 1 ‘not at all like me’ to 7 ‘very much like me.’

*Perceived gender discrimination (Sample 4)*. We used one item to gauge perceptions of gender discrimination (‘Please indicate how much you think you personally experience discrimination due to your gender’), from 1 ‘not at all’ to 10 ‘very much.’

#### Incremental validity

*Neoliberal Beliefs Inventory (Sample 3)*. We included the 25-item Neoliberal Beliefs Inventory, which is comprised of four dimensions (system inequality, competition, personal wherewithal, government interference; ω = 0.92; Bay-Cheng et al., 2015; e.g., ‘The government often hurts individual ambition when it interferes’). Because certain items mention affirmation action and this concept is not widespread in France, we added a brief definition for participants who might be unfamiliar with the term.

#### Additional measure

*Social desirability (Sample 3)*. We assessed social desirability using 17 items from Juhel and Rouxel’s (2005) scale (ω = 0.84). We excluded two items: one that focused on personal control, because we felt it was too similar to items on the NOQ, and one that seemed highly redundant with another item (we excluded ‘I’m always polite, even with unpleasant people’ and included ‘I’m always polite’). The scale has two subscales, namely self-deception and hetero- (other-) deception. Following Tournois, Mesnil and Kop.’s (2000) instructions, participants rated each item on a scale ranging from 1 (‘entirely false’) to 7 (‘entirely true’).

## Results

### Item Analyses (Sample 1)

Inspection of descriptive statistics, including response distributions and item-total correlations (following Bay-Cheng et al.’s [2015] cutoff of *r* < .30) led us to remove 4 items, which left 64 items for the Exploratory Factor Analysis.^3^

### Exploratory Factor Analysis (Sample 1)

As a first step, sampling adequacy was assessed with the Kaiser-Meyer-Olkin’s test (MSA = .91). We assessed whether the correlation matrix differed significantly from a matrix including only null correlations by using Bartlett’s test of sphericity, which was significant (*p* <.001). Both indices were satisfactory (Tabachnick & Fidell, 2007), so there were no concerns about proceeding with the EFA.

We followed Costello and Osborne’s (2005) recommendations to determine the number of factors to retain by relying primarily on the scree-test and conducting several EFAs to identify the most appropriate structure. The fa.parallel function in R was used for factor retention. We specified principal axis factoring for extraction with an oblimin rotation, because the factors were expected to correlate. This analysis suggested that eight factors could be retained, but inspection of the Scree Plots revealed that the ‘break’ appeared after the seventh factor and that only seven factors surpassed the threshold for simulated and resampled data.

However, we did not find this structure to be entirely satisfactory because some of the factors seemed to lack conceptual clarity and distinctness. There was too much conceptual overlap between some factors to offer a clear interpretation of the factors. Therefore we also tested five- and six-factor structures to see whether they would summarize the data in a more clear-cut manner. We rejected the five-factor solution because only two items loaded on the fifth factor, and it is recommended to have at least three items per factor (Costello & Osborne, 2005). At this stage of scale development, we thus settled on a six-factor solution, which seemed most appropriate because it generated more interpretable factors than the seven-factor solution, and then proceeded to eliminate items based on conceptual considerations, factor loadings <.30 (Costello & Osborne, 2005), and cross-loadings >.10 (Bay-Cheng et al., 2015). We followed an iterative process of item elimination and factor analyses (Costello & Osborne, 2005). This led ultimately to a four-factor solution that was comprised of 32 items (see Table 2 and Supplementary Material 2 for the French version and Supplementary Material 3 for the 5–, 6–, and 7-factor solutions).

**Table 2:** EFA (Sample 1) and CFA (Sample 2) Results.


	EFA results					CFA RESULTS
	
	FACTOR LOADINGS				COMMUNALITIES	FACTOR LOADINGS

NOQ ITEMS	1	2	3	4		

**Factor 1: Competitiveness**						

(S) I think that competition is inevitable	**0.69**	–0.04	0.09	–0.05	0.45	0.55

Competition is what allows society to be efficient	**0.69**	0.06	–0.01	0.11	0.60	0.84

(S) Competition is the best way to spot talented people	**0.83**	–0.03	–0.02	0.00	0.66	0.80

(S) Competition is the best way to encourage us to do our best	**0.77**	0.04	0.02	–0.04	0.60	0.81

(X-) We need more competitiveness in society	**0.50**	0.03	–0.08	0.24	0.43	–

**Factor 2: Individual self-regulation**						

It is only because we lack courage or self-confidence that we do not seize the opportunities offered to us	–0.04	**0.45**	0.06	0.12	0.25	0.60

With proper planning, all our goals are achievable	–0.01	**0.60**	–0.04	0.12	0.38	0.66

The secret of success is to know yourself well	0.09	**0.48**	0.15	–0.20	0.31	0.64

When you encounter difficulties, the first thing to do is to question yourself	0.05	**0.41**	0.16	–0.08	0.24	0.42

(S) It is mainly by working on ourselves that we can change the circumstances of our lives	0.02	**0.60**	0.07	0.01	0.40	0.67

(S) With the right kind of motivation you can do anything	0.04	**0.70**	–0.03	–0.04	0.49	0.72

There is always something positive to be gained from any situation, even the worst	–0.06	**0.51**	0.05	–0.09	0.25	0.52

(S) We can find solutions to all of the obstacles we encounter in life	–0.05	**0.53**	0.05	0.09	0.31	0.63

Rather than trying to change society, everyone should work on themselves	0.02	**0.45**	0.09	0.11	0.29	0.52

To be happy, we simply need to focus on the positive	0.04	**0.49**	–0.03	–0.08	0.24	0.50

When we are not going well, we only need to change our perspective on the situation to feel better	0.00	**0.54**	–0.15	0.11	0.29	0.60

(X-) It is up to each person to adapt to all situations	0.08	**0.45**	0.05	0.12	0.30	–

**Factor 3: Relational detachment**						

When a relationship does not benefit me, I prefer to put an end to it	0.05	00	**0.53**	–0.05	0.28	0.43

Depending on others makes us vulnerable	0.19	–0.01	**0.46**	0.01	0.28	0.54

(S) It is important not to depend on other people	0.06	0.11	**0.50**	–0.03	0.32	0.67

(S) When people hold us back from our goals, it’s best to let them go	–0.02	0.03	**0.71**	0.10	0.56	0.53

It is better to part with people who waste our time	0.00	–0.05	**0.72**	0.09	0.53	0.55

(S) We should do more to make our own personal choices without being influenced by other people	–0.05	0.23	**0.47**	–0.17	0.32	0.57

**Factor 4: Public divestment**						

(S) Lowering taxes for the wealthiest allows them to invest and therefore to create wealth for all	0.12	0.12	–0.08	**0.47**	0.32	0.60

A public service like Pôle Emploi* should be managed by a private company rather than by the State	–0.01	–0.04	0.13	**0.56**	0.33	0.52

The State must let business owners manage their companies as they wish	0.08	0.06	0.00	**0.48**	0.29	0.50

Ensuring that everyone has the same economic resources is not the responsibility of the State	0.17	0.19	–0.08	**0.43**	0.36	0.61

The State must guarantee the freedom of citizens rather than equality between citizens	0.12	0.05	0.08	**0.42**	0.28	0.46

The State should spend less money on public services	–0.01	–0.05	0.03	**0.74**	0.54	0.63

Reducing France’s debt must be a top priority	0.09	0.08	0.11	**0.37**	0.24	0.44

(S) Helping people in difficulty is the job of non-profit organizations, not the government	0.06	0.07	–0.05	**0.62**	0.44	0.59

(S) Privatizing public services would make them more efficient	0.01	0.00	0.12	**0.65**	0.47	0.58

	Pct. of variance explained					

	9%	11%	7%	10%		


*Note*: Items preceded by ‘(X-)’ were removed during the CFA. The items were originally presented in French for Samples 1–3; we translated them for the purpose of this article and so that the NOQ could be administered to English speakers. Items preceded by ‘(S)’ were those selected for the NOQ-S in Sample 4 and administered in English. * Pôle emploi is a French public agency which registers unemployed people, helps them find jobs, and provides them with financial aid.

The four-factor solution explained 38% of the overall variance in responses. Correlations among the four factors were all positive and significant (see Table 3), suggesting that the oblimin rotation was warranted. We labeled the four factors as follows: (1) Competitiveness (how much people value competition for themselves and for society); (2) Individual self-regulation (how much people focus on personal responsibility and self-control); (3) Relational detachment (how much people seek independence and avoid social interdependence); and (4) Public divestment (how much people desire the state [or government] to disengage from social and economic life; see Table 2 and Tables 3, 4, 5 and 6 for the means and standard deviations for the NOQ and its subscales in each sample).

**Table 3:** Means, Standard Deviations, and Correlations for Sample 1.


VARIABLE (RANGE)	*M*	*SD*	1	2	3	4	5	6	7	8

1. NOQ (1–7)	4.43	0.68								

2. COMPETITIVE	3.94	1.20	0.73***							

3. IND SELF-REG	4.83	0.74	0.71***	0.36***						

4. DETACHMENT	5.06	0.89	0.56***	0.23***	0.38***					

5. DIVESTMENT	3.78	1.06	0.76***	0.55***	0.27***	0.23***				

6. ESJ (1–7)	3.62	0.74	0.56***	0.49***	0.26***	0.15***	0.63***			

7. FWS (1–7)	4.71	0.93	0.55***	0.38***	0.52***	0.28***	0.40***	0.35***		

8. PGI (1–7)	4.91	0.76	0.40***	0.20***	0.51***	0.37***	0.13**	0.07	0.39**	

9. Pol-OR (1–9)	5.08	1.85	0.40***	0.33***	0.15**	0.12*	0.51***	0.46***	0.27***	–0.01


*Note*: *M* and *SD* are used to represent mean and standard deviation, respectively. * indicates *p* < .05. ** indicates *p* < .01 and *** indicates *p* < .001. All data from Sample 1 relies on the 32-item version of the NOQ. COMPETIVE = NOQ Competitiveness subscale. IND SELF-REG = NOQ Individual Self-regulation subscale. DETACHMENT = NOQ Relational Detachment subscale. DIVESTMENT = NOQ Public Divestment subscale. ESJ = Economic System Justification scale. FWS = Free Will Scale. PGI = Personal Growth Initiative scale. Pol-OR = Political Orientation ranging from ‘extreme left’ to ‘extreme right.’

**Table 4:** Means, Standard Deviations, and Correlations for Sample 2.


VARIABLE (RANGE)	*M*	*SD*	1	2	3	4	5	6	7	8	9

1. NOQ (1–7)	4.47	0.68									

2. COMPETITIVE	4.25	1.11	0.66***								

3. IND SELF-REG	4.81	0.81	0.76***	0.37***							

4. DETACHMENT	5.08	0.87	0.67***	0.35***	0.50***						

5. DIVESTMENT	3.73	0.98	0.68***	0.43***	0.24***	0.21***					

6. GSJ (1–7)	3.09	0.96	0.21***	0.22***	0.17***	–0.08	0.30***				

7. SOC CON (0–100)	54.22	17.02	0.34***	0.29***	0.29***	0.13**	0.26***	0.19***			

8. SILVER (1–7)	4.48	0.75	0.32***	0.24***	0.44***	0.22***	0.04	0.17**	0.17**		

9. SUM_LOC (11–44)	27.13	5.16	0.33***	0.25***	0.34***	0.15**	0.19***	0.22***	0.17***	0.18**	

10. Pol-OR (1–9)	5.00	1.78	0.34***	0.25***	0.13**	0.14**	0.42***	0.10*	0.36***	0.02	0.15**


*Note*: *M* and *SD* are used to represent mean and standard deviation, respectively. * indicates *p* < .05. ** indicates *p* < .01 and *** indicates *p* < .001. COMPETIVE = NOQ Competitiveness subscale. IND SELF-REG = NOQ Individual Self-regulation subscale. DETACHMENT = NOQ Relational Detachment subscale. DIVESTMENT = NOQ Public Divestment subscale. GSJ = General System Justification scale. SOC CONSERV = Social Conservatism scale. SILVER = Silver Lining scale. SUM-LOC = Locus of Control Scale. Pol-OR = Political Orientation ranging from ‘extreme left’ to ‘extreme right.’^4^

**Table 5:** Means, Standard Deviations, and Correlations for Sample 3.


VARIABLE (RANGE)	*M*	*SD*	1	2	3	4	5	6	7	8	9	10

1. NOQ (1–7)	4.46	0.67										

2. COMPETITIVE	3.98	1.24	0.70***									

3. IND SELF-REG	4.84	0.81	0.77***	0.35***								

4. DETACHMENT	5.31	0.89	0.58***	0.23***	0.45***							

5. DIVESTMENT	3.65	0.92	0.73***	0.55***	0.32***	0.15*						

6. NBI (1–7)	4.04	0.85	0.60***	0.47***	0.40***	0.31***	0.57***					

7. FEM (1–7)	3.71	1.22	0.05	0.08	0.12	–0.10	0.02	–0.08				

8. SDO (1–7)	2.85	0.98	0.35***	0.35***	0.08	–0.05	0.59***	0.56***	–0.10			

9. DES (1–7)	4.08	0.76	0.11	0.07	0.14*	–0.05	0.06	0.07	–0.07	0.01		

10.SUM_AC (0–25)	5.64	3.94	0.05	0.02	0.08	0.07	–0.08	–0.14*	0.51***	–0.22**	–0.04	

11.OR Pol (1–9)	4.97	1.60	0.33***	0.25***	0.17*	0.10	0.45***	0.50***	–0.14*	0.43***	0.05	–0.18*


*Note*: *M* and *SD* are used to represent mean and standard deviation, respectively. * indicates *p* < .05. ** indicates *p* < .01 and *** indicates *p* < .001. COMPETIVE = NOQ Competitiveness subscale. IND SELF-REG = NOQ Individual Self-regulation subscale. DETACHMENT = NOQ Relational Detachment subscale. DIVESTMENT = NOQ Public Divestment subscale. NBI = Neoliberal Beliefs Inventory. FEM = Feminist identification scale. SDO = Social Dominance Orientation scale. DES= Social Desirability scale. SUM_CA = Collective Action scale. Pol-OR = Political orientation ranging from ‘extreme left’ to ‘extreme right.’

**Table 6:** Means, Standard Deviations, and Correlations for Sample 4.


VARIABLE (RANGE)	*M*	*SD*	1	2	3	4	5	6	7	8	9	10

1. NOQ-S (1–7)	4.22	0.79										

2. COMPETITIVE	4.53	1.34	0.73***									

3. IND SELF-REG	4.60	1.32	0.76***	0.38***								

4. DETACHMENT	5.18	1.01	0.48***	0.14**	0.35***							

5. DIVESTMENT	2.58	1.13	0.57***	0.29***	0.24***	–0.05						

6. GSJ (1–9)	3.50	1.33	0.41***	0.35***	0.22***	–0.14**	0.60***					

7. ESJ (1–9)	4.14	1.16	0.56***	0.45***	0.30***	0.01	0.67***	0.68***				

8. FUTUR (1–7)	4.82	0.84	0.11*	0.07	0.07	0.19***	–0.08	–0.10*	–0.07			

9. Pol-OR (1–11)	4.26	1.91	0.43***	0.35***	0.22***	0.02	0.54***	0.49***	0.64***	0.03		

10. SOC_CONS (1–11)	3.74	2.05	0.41***	0.30***	0.19***	0.03	0.54***	0.50***	0.59***	–0.04	0.81	

11. ECO_CONS (1–11)	4.65	2.11	0.47***	0.36***	0.25***	0.03	0.57***	0.49***	0.58***	0.00	0.77	0.64***


*Note*: *M* and *SD* are used to represent mean and standard deviation, respectively. * indicates p < .05. ** indicates p < .01 and *** indicates p < .001. NOQ-S = short version of the NOQ (12 items). COMPETITIVE = NOQ-S Competitiveness subscale. IND SELF-REG = NOQ-S Individual Self-regulation subscale. DETACHMENT = NOQ-S Relational Detachment subscale. DIVESTMENT = NOQ-S Public Divestment subscale. GSJ = General System Justification scale. ESJ = Economic system justification scale. FUTUR = CFC-14 scale assessing orientation toward the future. Pol-OR = Political Orientation ranging from ‘extremely liberal’ to ‘extremely conservative.’ SOC CONS = Social Conservatism item. ECO_CONS = Economic conservatism item.

### Confirmatory Factor Analysis (Sample 2)

Using the Lavaan package in R, we used the data from Sample 2 to test whether the four-factor solution with 32 items obtained with the EFA provided a good fit to the new data. No item exhibited a skewness of >1 and a kurtosis >1.5, indicating correct normality to proceed with the Maximum Likelihood estimation method (Harrington, 2009). Base on the EFA results, we specified which items would load onto each factor, and the factors were allowed to correlate with one another. We relied on CFI ≥ .90 and RMSEA and SRMR ≤ .10 as criteria for acceptable fit because there were fewer than 500 participants (Weston & Gore, 2006).^5^

Results indicated that the four-factor structure provided an acceptable fit for the data with respect to two indices (RMSEA = .05 and SRMR = .06) but not the third (CFI = .87). Inspection of factor loadings (cutoff set up for items ≤.40) and modification indices led us to remove two items and to allow three error variances to correlate: (1) between the items ‘To be happy, we simply need to focus on the positive’ and ‘When we are not going well, we only need to change our perspective on the situation to feel better’; (2) between the items ‘A public service like Pôle Emploi should be managed by a private company rather than by the State’ and ‘Privatizing some public services would make them more efficient’; and (3) between the items ‘When people hold us back from our goals, it’s best to let them go’ and ‘It is better to part with people who waste our time.’^6^ This yielded the best fit, RMSEA = .05, SRMR = .06, and CFI = .91 (see Table 2 for CFA results and Table 6 for descriptive information about the scale and its subscales).

Finally, we tested another unidimensional model to see if it would fit the data better than the modified four-factor solution. Results revealed that the fit was unsatisfactory, RMSEA = .09, SRMR = .10, and CFI = .59, indicating that the four-factor solution was indeed a better model.

### Internal Consistency and Sensitivity (Samples 1–4)

To assess the internal consistency of the NOQ and its subscales, we relied on two complementary indices. We utilized the McDonald’s Omega reliability coefficient and the split-half method (calculating a corrected correlation coefficient between odd and even items; Broc et al., 2016). The discriminatory power of the NOQ was assessed in terms of Ferguson Delta, which ranges from 0 to 1 (the closer to 1 the more successfully the scale discriminates among people). Results are summarized in Table 7.

**Table 7:** NOQ Descriptive Information, Internal Consistency, and Sensitivity.


	DESCRIPTIVE INFORMATION				OMEGA COEFFICIENT (*ω*)			
	
	SAMPLE 1 (*N* = 580)	SAMPLE 2 (*N* = 476)	SAMPLE 3 (*N* = 235)	SAMPLE 4 (*N* = 449)	SAMPLE 1 (*N* = 580)	SAMPLE 2 (*N* = 476)	SAMPLE 3 (*N* = 235)	SAMPLE 4 (*N* = 449)

	*M* (SD)	*M* (SD)	*M* (SD)	*M* (SD)	(*ω*)	(*ω*)	(*ω*)	(*ω*)

**NOQ**	4.43 (0.68)	4.47 (0.68)	4.46 (0.67)	4.22 (0.79)	0.90	0.89	0.89	0.75

**Competitiveness**	3.94 (1.20)	4.25 (1.11)	3.98 (1.24)	4.53 (1.34)	0.85	0.84	0.85	0.82

**Individual Self-Regulation**	4.83 (0.74)	4.81 (0.81)	4.84 (0.81)	4.60 (1.32)	0.83	0.86	0.86	0.77

**Relational Detachment**	5.06 (0.89)	5.08 (0.87)	5.31 (0.89)	5.18 (1.01)	0.76	0.74	0.77	0.54

**Public Divestment**	3.78 (1.06)	3.73 (0.98)	3.65 (0.92)	2.58 (1.13)	0.83	0.80	0.79	0.72

	**Corrected Spearman-Brown correlation between odd and even items**				**Ferguson Delta**			

	0.93	0.87	0.86	0.74	0.99	0.99	0.99	0.98


*Note*: All data from Sample 1 relies on the 32-item version of the NOQ and data from Sample 4 rely on the 12-item NOQ-S (see Table 2). The NOQ is a 7-point scale ranging from 1 ‘strongly disagree’ to 7 ‘strongly agree.’

### Validity (Samples 1–4)

NOQ scores correlated positively and significantly with economic system justification, *r* = 0.56, *p* < .001 (Sample 1), *r* = 0.56, *p* < .001 (Sample 4; NOQ-S), general system justification, *r* = 0.21, *p* < .001 (Sample 2), *r* = 0.41, *p* < .001 (Sample 4; NOQ-S), belief in free will, *r* = 0.55, *p* < .001, personal growth initiative, *r* = 0.40, *p* < .001, internal locus of control, *r* = 0.33, *p* < .001, silver lining scores, *r* = 0.32, *p* < .001, being future-oriented, *r* = 0.11, *p* = .024, social conservatism, *r* = 0.34, *p* < .001 (Sample 2), *r* = 0.41, *p* < .001 (Sample 4; NOQ-S), economic conservatism, *r* = 0.47, *p* < .001, and social dominance orientation, *r* = 0.35, *p* < .001.^7^ The NOQ also correlated positively and significantly with the NBI, but not to the point of being redundant, *r* = 0.60, *p* < .001, and it was unrelated to social desirability, *r* = 0.11, *p* = .096.

NOQ scores were correlated with political orientation in all 4 samples, *r* = 0.42, *p* < .001 (Sample 1), *r* = 0.33, *p* < .001 (Sample 2), *r* = 0.33, *p* < .001 (Sample 3), *r* = 0.43, *p* < .001 (Sample 4; NOQ-S). Thus, neoliberal ideology was more popular on the right than the left. Contrary to our expectations, the NOQ-S was unrelated to perceived gender discrimination among self-identified women, *r* = –0.08, *p* = .173. Moreover, NOQ scores were uncorrelated with feminist identification, *r* = 0.05, *p* = .445 and self-reported engagement in collective action on behalf of women, *r* = 0.05, *p* = .483 (see Tables 3, 4, 5 and 6 for the correlations).^8^

In terms of demographic variables, subjective social class was the only one that correlated significantly with the NOQ in the French samples, *r* = 0.13, *p* = .002 (Sample 1), *r* = 0.16, *p* < .001 (Sample 2), *r* = 0.24, *p* < .001 (Sample 3). The higher participants placed themselves on the socio-economic ladder, the more they endorsed neoliberal ideology. There was no correlation with gender, *r* = 0.06, *p* = .186 (Sample 1), *r* = 0.06, *p* = .205 (Sample 2), or age, *r* = –0.06, *p* = .184 (Sample 1), *r* = –0.00, *p* = .984 (Sample 2), *r* = –0.01, *p* = .846 (Sample 3). In the US student sample, the NOQ-S correlated negatively with gender identity, *r* = –0.20, *p* < .001 and sex, *r* = –0.17, *p* < .001 (Sample 4), indicating that men endorsed neoliberal ideology more than women did.

#### Incremental Validity

In Sample 3 (all female), we predicted that NOQ scores would predict feminist identification, engagement in CA, and social dominance orientation even after adjusting for NBI scores. We conducted hierarchical regressions in which the NBI was entered as a predictor in step 1, and then NOQ was added as predictor in step 2. NBI and NOQ scores were standardized.

NBI scores did predict feminist identification negatively and significantly in step 1, *b* = –0.23, *SE* = .093, *t*(232) = –2.53, 95% CI [–0.42, –0.05], *p* = .012, *R²* = .03. In step 2, NBI continued to predict feminist identification, *b* = –0.40, *SE* = .104, *t*(231) = –3.90, 95% CI [–0.61, –0.20], *p* < .001, *η^2^_p_ =* .06, and, importantly, NOQ also predicted feminist identification, but in the opposite direction, *b* = 0.31, *SE* = .104, *t*(231) = 2.98, 95% CI [0.11, 0.52], *p* = .003, *η^2^_p_ =* .04, *R²* = .06, *ΔR²* = .04, *ΔF* = 8.90, *p* = .003. We explore this unexpected result in more detail below.

In step 1, the NBI negatively predicted self-reported engagement in feminist CA, *b* = –0.67, *SE* = .300, *t*(231) = –2.23, 95% CI [–1.26, –0.08], *p* = .027, *R²* = .02. This was also true in step 2, *b* = –1.17, *SE* = .304, *t*(228) = –3.86, 95% CI [–1.77, –0.57], *p* < .001, *η^2^_p_ =* .06, and NOQ scores again predicted engagement in the opposite direction, *b* = 0.72, *SE* = .303, *t*(228) = 2.39, 95% CI [0.13, 1.32], *p* = .018, *η^2^_p_ =* .02, *R²* = .04, *ΔR²* = .02, *ΔF* = 4.20, *p* = .041.^9^

The NBI predicted social dominance orientation in step 1, *b* = 0.67, *SE* = .061, *t*(233) = 10.88, 95% CI [0.55, 0.79], *p* < .001, *R²* = .34, and in step 2, *b* = 0.60, *SE* = .070, *t*(232) = 8.63, 95% CI [0.46, 0.74], *p* < .001, *η^2^_p_ =* .24. However, NOQ scores did not predict social dominance orientation after adjusting for NBI scores, *b* = –0.05, *SE* = .070, *t*(232) = –0.73, 95% CI [–0.19, 0.09], *p* = .466, *R²* = .34, *ΔR²* = .00, *ΔF* = 0.53, *p* = .466.

We conducted additional exploratory analyses to understand the surprising positive association between the NOQ and feminist identification and collective action after adjusting for NBI scores. We suspected that NOQ scores might interact with participants’ political orientation to predict these outcomes, in part because of the emergence of neoliberal feminism (Rottenberg, 2018). Indeed, if women, especially those on the right, endorse a form of neoliberal feminism, then we would expect a positive correlation between neoliberal ideology and feminist identification. These women might conceive of feminism primarily as a way of defending women’s upward mobility within the capitalist system, and might be attracted to the individual self-regulation and relational detachment aspects of neoliberal ideology, as assessed by the NOQ, as means of achieving upward mobility. For leftists, however, it may be that the feminist label constitutes a politicized identity, with an emphasis on collective (rather than individual) action. Thus, we explored the possibility that NOQ scores would predict feminist identification positively for women on the right, but negatively for women on the left.

We first conducted a regression analysis in which NOQ scores, political orientation (both standardized), as well as their interaction were entered as predictors of feminist identification. First, there was a negative main effect of political orientation, *b* = –0.22, *SE* = .086, *t*(217) = –2.50, 95% CI [–0.39, –0.05], *p* = .013, *η^2^_p_ =* .03, meaning that feminist identification was stronger on the left than the right in general. After adjusting for political orientation, NOQ scores were marginally but positively associated with feminist identification, *b* = 0.16, *SE* = .087, *t*(217) = 1.87, 95% CI [–0.01, 0.33], *p* = .063, *η^2^_p_ =* .02. Importantly, the interaction between political orientation and NOQ scores was significant, *b* = 0.16, *SE* = .070, *t*(217) = 2.33, 95% CI [0.02, 0.30], *p* = .021, *η^2^_p_ =* .02. Inspection of simple effects (at +/– 1 standard deviation from the mean score on political orientation) revealed that for rightist women, the effect of NOQ scores on feminist identification was significant and positive, *b* = 0.33, *SE* = .123, *t*(217) = 2.64, 95% CI [0.08, 0.57], *p* = .009, *η^2^_p_ =* .03. For leftist women, the effect of NOQ on feminist identification was not significant, *b* = –0.00, *SE* = .099, *t*(217) = –0.01, 95% CI [–0.20, 0.19], *p* = .991.

We conducted parallel analyses for self-reported engagement in feminist CA and also observed a significant negative main effect of political orientation, *b* = –0.73, *SE* = .279, *t*(216) = –2.62, 95% CI [–1.28, –0.18], *p* = .001, *η^2^_p_ =* .03. After adjusting for political orientation, the effect of NOQ was not significant, *b* = 0.45, *SE* = .281, *t*(216) = 1.593, 95% CI [–0.11, 1.00], *p* = .113. However, the interaction was significant, *b* = 0.71, *SE* = .227, *t*(216) = 3.12, 95% CI [0.26, 1.16], *p* = .002, *η^2^_p_ =* .04. The more right-leaning women endorsed neoliberal ideology, the more they reported engaging in feminist CA, *b* = 1.16, *SE* = .399, *t*(216) = 2.90, 95% CI [0.37, 1.94], *p* = .004, *η^2^_p_ =* .04. The simple effect was non-significant among left-leaning women, *b* = –0.26, *SE* = .318, *t*(216) = –0.82, 95% CI [–0.89, 0.37], *p* = .411.

Finally, inspection of correlations between the NOQ and the NBI subscales revealed no major concerns about redundancy. Even for the dimension of competition, the two subscales were highly correlated at *r* = 0.71, *p* < .001, but they were not so highly correlated as to be considered identical (see Table 8 for the correlations between the NOQ and NBI subscales).

**Table 8:** Correlations between the NOQ and the NBI subscales.


VARIABLE (RANGE)	*M*	*SD*	1	2	3	4	5	6	7

1. NOQ-COMP	3.98	1.24							

2. NOQ- REG	4.84	0.81	0.35***						

3. NOQ-DETACH	5.31	0.89	0.23***	0.45***					

4. NOQ-DIVEST	3.65	0.92	0.54***	0.32***	0.14*				

5. NBI-SI	3.81	1.13	0.30***	0.20**	0.24***	0.47***			

6. NBI-CO	4.22	0.89	0.71***	0.35***	0.28***	0.44***	0.36***		

7. NBI-PW	4.12	1.04	0.34***	0.46***	0.25***	0.42***	0.61***	0.48***	

8. NBI-GI	4.06	1.04	0.38***	0.38***	0.29***	0.59***	0.62***	0.40***	0.53***


*Note*: *M* and *SD* are used to represent mean and standard deviation, respectively. * indicates p < .05. ** indicates p < .01 and *** indicates p < .001. NOQ-COMP = NOQ Competitiveness subscale. NOQ-REG = NOQ Individual Self-regulation subscale. NOQ-DETACH = NOQ Relational Detachment subscale. NOQ-DIVEST = NOQ Public Divestment subscale. NBI-SI = NBI System Inequality subscale. NBI-CO = NBI Competition subscale. NBI-PW = NBI Personal Wherewithal subscale. NBI-GI = NBI Government Interference subscale.

## Discussion

Although social psychologists have begun to study neoliberal ideology in recent years, there is no clear-cut definition in the literature that is consensually shared (e.g., Birch, 2015). There is some disagreement about what the core (vs. peripheral) elements of neoliberal ideology are. When researchers seek to measure a complex, multifaceted concept such as neoliberalism, item construction is necessarily driven by the authors’ definition of the construct. Both the NBI and the ANAS include items tapping into perceptions of inequality and potential solutions to it, whereas we did not include such items—largely because we wanted to determine empirically whether support for neoliberalism is linked to acceptance of inequality (Azevedo et al., 2019). Another distinctive feature of the NOQ is its focus on the *entrepreneurial self*, which emphasizes individual self-regulation and cost-benefit calculations with respect to interpersonal relationships (Teo, 2018). Along these lines, we endeavored to develop a new and distinctive measure of neoliberal ideology that would incorporate personal as well as social values and that would be applicable to European as well as North American contexts.

We began by generating items based on the results of semi-structured interviews conducted in France (Girerd, Verniers & Bonnot, 2021). Next, we conducted factor analyses that yielded a 30-item instrument with four dimensions: competitiveness (as something beneficial and necessary), individual self-regulation (reflecting personal responsibility and self-transformation), relational detachment (a desire for autonomy and the application a cost-benefit analysis to interpersonal relations), and public divestment (support for government retrenchment from economic affairs).

In the present set of studies, we obtained support for the construct validity of the NOQ. Specifically, we observed significant and positive correlations between NOQ scores and NBI scores, economic and general system justification (in France and the US), economic conservatism, internal locus of control, personal growth initiative, future orientation, ‘silver lining’ assumptions, and social dominance orientation. Although these findings are correlational in nature, they are consistent with the theoretical proposition that neoliberal ideology is a system-justifying, hierarchy-enhancing belief system (Azevedo et al., 2019; Harvey, 2007; Monbiot, 2016). Neoliberal ideology (as measured with the NOQ) was positively rather than negatively correlated with social conservatism in two samples from France and the US. On the basis of these findings, we would not conclude that neoliberal ideology is a consistently libertarian orientation; rather, it was associated with the legitimation of social as well as economic aspects of the status quo (Azevedo et al., 2019). At the same time, it is true that NOQ scores were more strongly correlated with economic (vs. general) system justification. This was especially true in the French samples, which might suggest a minor cultural difference between France and the US.

Our findings are largely consistent with previous findings based on the NBI and ANAS. For one thing, NOQ scores were correlated with internal locus of control scores and with social dominance orientation. Interestingly, endorsing neoliberal ideology seems to be associated with a propensity to find ‘silver linings,’ a form of cognitive reappraisal that is expected to enhance individual well-being (Haga et al., 2009). However, other research has shown that by promoting competition and social disconnection, neoliberal ideology might in fact impede individual well-being (Becker et al., 2021). More research is needed at this stage to disentangle such seemingly contradictory findings.

Perhaps surprisingly, NOQ scores were not consistently associated with perceived gender discrimination, feminist identification, or self-reported engagement in feminist collective action among women in France. Furthermore, after adjusting for NBI scores, NOQ scores were positively correlated with feminist identification. Follow-up analyses suggested that this effect was driven by rightists and was non-significant among leftists. This is consistent with the idea that there is a form of neoliberal feminism in France that is grounded in women’s support for meritocratic individualism—rather than collective opposition to sexism (Bongiorno et al., 2021; Kim, Fitzsimons & Kay, 2018; Rottenberg, 2018). Consistent with perceived self-interest, NOQ scores were positively (but moderately) correlated with subjective social class in the French samples.

We consider these studies to have made a useful contribution to the study of neoliberal ideology in psychology. At the same time, much more research is needed to broaden our understanding of this ideology and how the NOQ relates to other psychological instruments. We note several limitations of our work so far. For instance, we note that the internal consistency for the relational detachment subscale was quite low in the US sample. Keeping in mind that a short version of the scale with only three items per dimension was administered to that sample, we see the importance of assessing the validity and reliability of the NOQ in other contexts. Moreover, we sought to test the incremental validity of the NOQ relative to the NBI, especially with respect to feminist identification, collective action, and social dominance orientation. However, NOQ scores were in fact unrelated to feminist identification (the NBI was also unrelated to feminist identification) and collective action. Thus, further demonstrations of the incremental validity of the NOQ would seem to be required. It is possible that by including more private elements of neoliberal ideology, the NOQ would be superior to the NBI and ANAS when it comes to predicting feelings of social disconnection for instance (Becker et al., 2021).

Future studies would do well to utilize experimental paradigms to identify contextual moderators of support for neoliberal ideology. For example, activating system justification motivation through manipulations of system criticism, or perceived system inescapability (Jost, 2020) would be hypothesized to increase NOQ scores. From a system justification perspective, it might be useful to study older populations, who came of age prior to the advent of the neoliberal status quo.

## Concluding Remarks

Neoliberalism is not merely a set of political and economic policies ushered in by the likes of Margaret Thatcher and Ronald Reagan (Harvey, 2007). Some forty years later, it is an ideology that continues to shape social and personal views about life, work, and family, among other things (Binkley, 2011a, 2011b; Monbiot, 2016; Teo, 2018). The belief system we have sought to conceptualize and measure is closely linked to the ideological defense of the societal status quo under capitalism in the 21^st^ century. In this sense, it clearly appears to function as a system-justifying ideology in both France and the US (Azevedo et al., 2019; Girerd, Verniers & Bonnot, 2021; Jost, 2020). Neoliberal ideology is not only a way of making sense of current social, economic, and political arrangements in the Western world; it also aims to legitimize and, in so doing, perpetuate those arrangements, even in the face of growing concerns about, among other things, socio-economic inequality and environmental sustainability.

## Data Accessibility statement

The data from Studies 1–4 may be accessed here: https://osf.io/e9q45/?view_only=ac8c00819af340d08f667d61dc285ed6.

## Additional File

The additional file for this article can be found as follows:

10.5334/irsp.663.s1 Supplementary materials. Supplementary Material 1 to 3.

## Footnotes

Because our items were administered as part of a much longer battery involving many different scales, it was necessary to construct a shortened version of the NOQ.

So that participants would not be left with negative feelings after answering this questionnaire, we administered a task in which they were asked to select their favorite pictures from an IAP selection that have been found to elicit feelings of joy (Lang, Bradley & Cuthbert, 2008). We also provided a list of websites in the debriefing section that participants could visit if they felt the need for psychological assistance.

In redoing the analyses, we realized that a fifth item should have been removed at this stage (item-total correlation of .29); it was instead removed at a later stage. Removing this item before running the EFA would not have changed the overall structure of the scale, but it would have led us to retain one more item in the final 4-dimension scale (33 instead of 32).

We also explored the association between neoliberal ideology and ‘Intensive Mothering Ideology.’ To the extent that people regard the primary responsibility of mothers as raising children who will become successful capitalist entrepreneurs, then a positive association would be expected (Milkie & Warner, 2014). In addition, to the extent that being a ‘good mother’ is seen as leveraging practices of personal control and individual self-regulation to be ‘in tune’ with one’s children, there is a fundamental compatibility between neoliberal ideology and intensive mothering ideology (Ennis, 2014; Hays, 1996; Verniers, Bonnot & Assilamehou-Kunz, 2022). According to Rottenberg (2018), the neoliberal woman is encouraged to seek the perfect, ‘happy’ balance involving intensive mothering, professional success, and personal fulfillment. Intensive Mothering Ideology (Sample 2) was assessed with the Measure of Intensive Mothering Ideology (i.e., MIMI; ω = 0.75; Loyal, Sutter Dallay & Rascle, 2021), which is comprised of 6 dimensions, namely essentialism, consuming fulfillment, child centrism, challenge, sacrifice, and stimulation. Exploratory analyses concerning intensive mothering ideology indicated a significant correlation with the NOQ, *r* = .34, *p* < .001 and most of its subscales, *r* = .09, *p* = .052, with the competitiveness subscale, *r* = .34, *p* <.001, with the self-regulation subscale, *r* = .39, *p* <.001, with the relational detachment subscale and *r* = .15, *p* = .001, with the Public Divestment subscale. Finally, the MIMI also correlated significantly with social conservatism, *r* = .25, *p* <.001, with the tendency to find silver linings, *r* = .17, *p* = .002, but it was unrelated to general system justification, *r* = –.04, *p* = .365 and to internal locus of control, *r* = –.01, *p* = .870.

Those findings could be replicated with larger samples to test how well the proposed model fits the data, this time relying on more stringent cut-off values (i.e., RMSEA ≤ .06, SRMR ≤ .08, CFI ≥ .95).

We think that the error variance of the first pair of items correlated because of the similarities between ‘simply need’ and ‘only need,’ the second pair because the two items share the idea of a transfer from the public sector to private companies and the third pair because in French the two items mention the same word ‘to part with’ (se séparer).

NOQ scores were correlated positively with both facets of social dominance orientation, namely group-dominance *r* = 0.39, *p* < .001 and opposition to equality *r* = 0.21, *p* = .002.

The results of Study 1 reported in the text are based on the 32-item scale that resulted from the EFA. We also re-ran the analyses with the 30-item version of the scale used in Studies 2 and 3, and the results were nearly identical.

In step 1 of the hierarchal regression when only the NBI was entered as the predictor of engagement in CA, five participants appeared as outliers (i.e., with the outliers function in R), three participants in step 2 when the NOQ was added as a predictor, and four participants in the regression analysis where the NOQ and political orientation were entered as predictors of engagement in CA, but the conclusions remained the same whether we including them or not, therefore we opted to report results that include them.
