# Supplementary material for: How Neoliberal are You? Development and Validation of the Neoliberal Orientation Questionnaire
Source: Int Rev Soc Psychol. 2023 Jul 19;36:11. doi: 10.5334/irsp.663 (PMC12372677; doi:10.5334/irsp.663)
Supplement: Supplementary materials. — Supplementary Material 1 to 3. [file irsp-36-663-s1.pdf]

Supplementary Material 1: Proximity with French political parties

	Lutte Ouvrière	NPA	PC	LFI	Générations-s	PS	Les Verts	LREM	MoDem	LR	Debout la France	RN	Other	None	Don't know
**Sample 1** (*N* = 580)	5 (0.9%)	8 (1.4%)	8 (1.4%)	34 (5.9%)	8 (1.4%)	50 (8.6%)	46 (7.9%)	55 (9.5%)	13 (2.2%)	31 (5.3%)	10 (1.7%)	47 (8.1%)	4 (0.7%)	213 (36.7%)	48 (8.3%)
**Sample 2** (*N* = 471)	10 (2.1%)	6 (1.3%)	4 (0.8%)	21 (4.5%)	2 (0.4%)	34 (7.2%)	39 (8.3%)	38 (8.7%)	7 (1.5%)	29 (6.2%)	5 (1.1%)	46 (9.8%)	3 (0.6%)	182 (38.6%)	45 (9.6%)
**Sample 3** (*N* = 230	4 (1.7%)	1 (0.4%)	3 (1.3%)	14 (6.1%)	2 (0.9%)	11 (4.8%)	27 (11.7%)	12 (5.2%)	0 (0%)	10 (4.3%)	2 (0.9%)	20 (8.7%)	0 (0%)	96 (41.7%)	28 (12.2%)

Notes. There were five missing data points in Sample 2 and in Sample 3 for this item. NPA = Nouveau Parti Anticapitaliste (New Anticapitalist Party). PC = Parti Communiste (Communist Party). LFI = La France Insoumise (The Unsubmissive France). PS = Parti Socialiste (Socialist Party). LREM = La République En Marche (The Republic in Motion). MoDem = Mouvement Démocrate (Democratic Movement). LR = Les Républicains (The Republicans). RN = Rassemblement National (National Rally).

Supplementary Material 2: items du NOQ en Français

Facteur 1 : Compétition

1/Je pense que la compétition est inévitable
2/ La compétition est ce qui permet à la société d'être efficace
3/ La compétition est le meilleur moyen pour repérer les talents
4/ La compétition est le meilleur moyen de nous pousser à faire de notre mieux
*Il faut plus de concurrence dans la société

Facteur 2 : Auto-régulation individuelle

5/ C'est uniquement parce que nous manquons de courage ou de confiance en soi que nous ne saisissons pas les opportunités qui s'offrent à nous
6/ En planifiant bien les choses, tous nos objectifs sont atteignables
7/ Le secret de la réussite est de bien se connaître soi-même
8/ Lorsqu'on rencontre des difficultés, la première chose à faire est de se remettre soi-même en question
9/ C'est surtout en faisant un travail sur soi qu'on peut modifier les conditions dans lesquelles on vit
10/ Avec de la motivation on peut tout faire
11/ Il y a toujours du positif à tirer de n'importe quelle situation, même les pires
12/ On peut trouver des solutions à tous les obstacles qu'on rencontre
13/ Plutôt que d'essayer de changer la société, chacun devrait travailler sur soi
14/ Pour être heureux, il suffit de se focaliser sur le positif
15/ Quand nous n'allons pas bien, il suffit de changer de perspective sur la situation pour que ça aille mieux
* C'est à chacun de s'adapter à toutes les situations

Facteur 3 : Détachement relationnel

16/ Lorsqu'une relation ne m'apporte rien, je préfère y mettre un terme
17/ Dépendre des autres nous rend vulnérables
18/ Il est important de ne pas dépendre d'autres personnes
19/ Lorsque des gens nous freinent dans nos objectifs, il est préférable de nous en séparer
20/ Il est préférable de se séparer des gens qui nous font perdre notre temps
21/ Nous devrions plus faire des choix qui nous correspondent vraiment sans se laisser influencer par les autres

Facteur 4 : Retrait de l'Etat

22/ Diminuer les impôts des plus fortunés leur permet d'investir et donc de créer de la richesse pour tous

23/ Un service public comme Pôle Emploi devrait être géré par une entreprise privée plutôt que par l'Etat
24/ L'État doit laisser les chefs d'entreprise gérer leurs sociétés comme ils veulent
25/ S’assurer que tout le monde ait les mêmes ressources économiques n’est pas du ressort de l’Etat
26/ L’Etat doit garantir la liberté des citoyens plutôt que l’égalité entre les citoyens
27/ L'Etat devrait mettre moins d'argent dans les services publics
28/ Réduire la dette de la France doit être une priorité absolue
29/ Venir en aide aux personnes en difficulté est le travail des associations et non celui de l’Etat
30/ Privatiser certains services publics permettrait de les rendre plus efficaces

*Items supprimés lors de l’analyse factorielle confirmatoire

Supplementary Material 3: EFA outputs for 7-factor, 6-factor and 5-factor structures

7-factor structure

		EFA results						
		Factor loadings						
**NOQ items**		**1**	**2**	**3**	**4**	**5**	**6**	**7**
1	It is only because we lack courage or self-confidence that we do not seize the opportunities offered to us	**0.48**	-0.07	0.07	0.08	-0.05	0.10	0.12
2	With proper planning, all our goals are achievable	**0.57**	-0.01	0.08	-0.08	0.08	0.05	0.05
3	The secret of success is to know yourself well	**0.40**	0.05	-0.14	0.03	0.20	0.12	-0.10
4	When you encounter difficulties, the first thing to do is to question yourself	**0.38**	0.05	-0.02	0.17	0.04	-0.04	-0.12
5	It is mainly by working on ourselves that we can change the circumstances of our lives	**0.58**	0.00	-0.03	0.07	0.04	0.07	0.08
6	With the right kind of motivation you can do anything	**0.66**	0.04	-0.03	-0.01	0.03	0.04	-0.12
7	If we don't succeed, it's because we make bad choices	0.27	0.13	0.17	-0.06	-0.03	0.03	0.20
9	Distinguishing myself from others through my personality is a priority to me.	0.11	0.05	0.05	-0.11	**0.51**	0.15	0.25
10	To succeed, you have to know how to sort out the people around you	0.04	-0.02	0.05	**0.34**	0.16	0.19	0.19
11	When a relationship does not benefit me, I prefer to put an end to it	-0.07	-0.01	0.00	0.33	0.28	0.13	0.07
13	It is important to regularly change our groups of friends in order to enrich ourselves on a personal level	-0.01	0.16	0.04	-0.04	0.18	0.02	**0.42**
14	I would be more successful in my endeavors without my close ones	-0.01	0.01	0.19	0.01	0.12	0.12	**0.46**
15	To fulfill our personal wishes, we shouldn't get too attached to other people	0.15	0.05	0.03	0.31	-0.05	0.11	**0.44**
16	Continuous improvement is a priority for me	0.22	0.18	-0.19	0.13	**0.44**	-0.15	-0.10
17	To give meaning to my life I constantly set new goals	0.23	0.22	-0.09	0.02	**0.40**	-0.03	-0.01
18	It is important to take risks in life	0.19	0.08	0.01	0.00	**0.38**	0.10	0.04
19	There is always something positive to be gained from any situation, even the worst	**0.47**	-0.09	-0.06	0.04	0.19	-0.10	-0.04

20	It is up to each person to adapt to all situations	**0.46**	0.06	0.11	0.04	-0.02	0.06	0.01
21	We can find solutions to all of the obstacles we encounter in life	**0.52**	-0.05	0.09	0.05	0.02	0.01	-0.04
22	Rather than trying to change society, everyone should work on themselves	**0.42**	-0.01	0.15	0.07	0.08	0.01	-0.04
23	To be happy, we simply need to focus on the positive	**0.47**	0.02	-0.12	-0.10	0.05	0.18	0.07
24	When we are not going well, we only need to change our perspective on the situation to feel better	**0.52**	0.01	0.01	-0.11	0.06	0.01	0.12
26	Controlling our emotions in all circumstances is crucial	0.18	0.20	-0.07	0.24	0.04	0.10	0.07
28	Depending on others makes us vulnerable	-0.01	0.19	-0.04	**0.63**	-0.13	0.07	0.05
29	It is important not to depend on other people	0.10	0.05	-0.03	**0.57**	-0.07	0.13	-0.07
30	In my opinion, freedom is not having any obligations	0.08	-0.01	0.03	-0.04	0.01	**0.56**	0.08
31	You are truly free when you can control your emotions	0.21	0.20	-0.09	0.14	0.09	0.13	0.23
32	In my opinion, to be free is to follow your own desires	0.14	-0.07	0.06	0.04	0.18	**0.45**	-0.15
33	Someone who is independent does not need government assistance to get by	0.19	0.19	0.21	0.10	-0.27	-0.01	0.13
34	To find a job, you have to accept relocation	0.16	0.24	0.07	0.03	0.01	0.02	0.11
35	It is important that our happiness does not depend on others	0.12	-0.01	-0.02	**0.51**	-0.01	0.17	-0.12
36	I think that competition is inevitable	-0.11	**0.70**	-0.02	0.03	0.14	0.02	-0.02
37	Competition is what allows society to be efficient	0.06	**0.67**	0.12	0.01	0.00	-0.03	0.03
38	Competition is the best way to spot talented people	-0.04	**0.79**	0.01	-0.01	0.00	0.05	0.00
39	We need more competitiveness in society	0.04	**0.53**	0.19	-0.07	-0.04	0.04	0.03
40	The competition is more a matter of one's mindset than of external pressures	0.26	0.19	0.05	0.04	-0.08	0.14	-0.02
41	Competition is the best way to encourage us to do our best	0.03	**0.73**	-0.01	0.03	-0.01	0.04	-0.04
42	Lowering taxes for the wealthiest allows them to invest and therefore to create wealth for all	0.14	0.16	**0.42**	-0.07	-0.02	-0.05	0.08
43	A public service like Pôle Emploi* should be managed by a private company rather than by the State	-0.05	0.00	**0.56**	0.03	0.08	0.04	0.06

44	The budget balance of France must be the main concern for the successive governments	0.09	0.08	0.34	-0.05	0.05	0.33	-0.22
45	The State must let business owners manage their companies as they wish	0.05	0.10	**0.44**	-0.03	-0.01	0.13	-0.01
46	The fact that salary differences be based on individual merit seems justified to me	0.24	0.30	0.11	0.15	-0.11	-0.21	-0.06
47	If one person possesses more than another, I assume that they deserve it	0.26	0.21	0.23	0.06	-0.02	-0.08	0.05
48	The important thing is not that everyone has the same resources, but rather that everyone is satisfied with what they have	**0.41**	0.06	0.09	0.09	-0.08	-0.07	-0.06
49	If people are not doing well financially, it is most often because they do not know how to handle their money	0.34	0.06	0.29	0.08	-0.16	-0.03	0.16
50	When people hold us back from our goals, it's best to let them go	-0.06	-0.10	0.20	**0.50**	0.33	0.09	0.11
51	It is better to part with people who waste our time	-0.12	-0.07	0.19	**0.48**	0.30	0.06	0.11
52	It's normal to socialize with certain people for the sole purpose of building a professional network	0.04	0.21	0.10	0.15	0.29	-0.04	0.20
53	We should make more choices that really correspond to us without being influenced by others	0.16	-0.10	-0.04	**0.40**	0.20	0.05	-0.16
54	To be independent is to have no obligation toward anyone else	0.03	0.11	-0.01	0.09	-0.04	0.62	0.02
55	To be free is to have nothing to ask of anyone	0.02	0.10	-0.04	0.17	-0.08	**0.63**	0.06
56	One should not give up personal wishes for other people	0.07	-0.12	0.17	0.21	0.15	**0.34**	-0.10
57	You should never rest on your laurels, but always seek to improve	0.14	0.08	0.01	0.26	**0.42**	-0.11	-0.28
58	It is more important to always strive for improvement than to be satisfied with a single success.	0.18	0.13	0.07	0.22	0.33	-0.14	-0.29
59	What gives meaning to one's life is to have clear personal goals.	0.28	0.08	0.08	0.10	0.34	0.03	-0.21
60	Multiplying new experiences is more important than keeping your habits	0.13	0.13	-0.01	0.05	**0.54**	0.02	0.03
61	Ensuring that everyone has the same economic resources is not the responsibility of the State	0.22	0.21	**0.37**	0.03	-0.03	-0.20	0.13
62	Higher education must be used to select the best and brightest for the job market	0.15	0.34	0.26	-0.04	0.00	-0.04	0.11
63	The State must guarantee the freedom of citizens rather than equality between citizens	0.08	0.17	**0.32**	0.15	-0.10	0.02	0.14
64	The State should spend less money on public services	-0.03	0.06	**0.67**	-0.03	-0.01	0.03	0.07
65	Reducing France's debt must be a top priority	0.03	0.11	**0.51**	-0.04	0.01	0.22	-0.28

66	People who invest their money in a company should be rewarded more than those who work there	-0.05	0.29	**0.34**	-0.17	0.08	0.07	0.21
67	Helping people in difficulty is the job of non-profit organizations, not the government	0.12	0.10	**0.52**	0.00	-0.09	-0.03	0.21
68	Privatizing public services would make them more efficient	0.00	0.05	**0.67**	0.12	0.01	-0.06	-0.09
		Pct. of variance explained						
		8%	7%	6%	5%	4%	4%	3%

6-factor structure

		EFA results					
		Factor loadings					
	**NOQ items**	**1**	**2**	**3**	**4**	**5**	**6**
1	It is only because we lack courage or self-confidence that we do not seize the opportunities offered to us	**0.46**	-0.06	0.11	-0.05	0.17	0.07
2	With proper planning, all our goals are achievable	**0.58**	-0.01	0.09	0.00	0.01	0.08
3	The secret of success is to know yourself well	**0.42**	0.05	-0.15	0.20	0.10	-0.01
4	When you encounter difficulties, the first thing to do is to question yourself	**0.35**	0.06	0.00	0.19	0.04	-0.13
5	It is mainly by working on ourselves that we can change the circumstances of our lives	**0.57**	0.02	0.00	0.04	0.12	0.07
6	With the right kind of motivation you can do anything	**0.66**	0.04	-0.01	0.05	0.04	-0.10
7	If we don't succeed, it's because we make bad choices	0.26	0.13	0.19	-0.13	0.03	0.17
9	Distinguishing myself from others through my personality is a priority to me.	0.18	0.02	0.00	0.26	0.00	**0.44**
10	To succeed, you have to know how to sort out the people around you	0.02	0.00	0.06	0.26	0.34	0.20
11	When a relationship does not benefit me, I prefer to put an end to it	-0.07	0.00	-0.01	**0.41**	0.24	0.15

13	It is important to regularly change our groups of friends in order to enrich ourselves on a personal level	-0.01	0.15	0.05	-0.01	0.00	**0.45**
14	I would be more successful in my endeavors without my close ones	-0.01	0.01	0.20	-0.06	0.12	**0.46**
15	To fulfill our personal wishes, we shouldn't get too attached to other people	0.09	0.08	0.10	-0.01	0.31	**0.31**
16	Continuous improvement is a priority for me	0.23	0.18	-0.21	**0.51**	-0.15	0.05
17	To give meaning to my life I constantly set new goals	0.26	0.21	-0.12	**0.37**	-0.08	0.14
18	It is important to take risks in life	0.23	0.06	-0.02	0.29	0.02	0.19
19	There is always something positive to be gained from any situation, even the worst	**0.47**	-0.09	-0.05	0.22	-0.10	0.01
20	It is up to each person to adapt to all situations	**0.45**	0.07	0.14	-0.01	0.09	-0.01
21	We can find solutions to all of the obstacles we encounter in life	**0.51**	-0.04	0.12	0.06	0.04	-0.04
22	Rather than trying to change society, everyone should work on themselves	**0.41**	-0.01	0.17	0.13	0.03	-0.02
23	To be happy, we simply need to focus on the positive	**0.49**	0.01	-0.12	-0.08	0.13	0.11
24	When we are not going well, we only need to change our perspective on the situation to feel better	**0.52**	0.00	0.03	-0.06	-0.04	0.14
26	Controlling our emotions in all circumstances is crucial	0.15	0.22	-0.05	0.15	0.23	0.05
28	Depending on others makes us vulnerable	-0.09	0.22	0.04	0.25	**0.40**	-0.08
29	It is important not to depend on other people	0.03	0.08	0.02	0.30	**0.42**	-0.15
30	In my opinion, freedom is not having any obligations	0.14	-0.03	0.01	-0.13	**0.49**	0.13
31	You are truly free when you can control your emotions	0.19	0.21	-0.08	0.06	0.20	0.22
32	In my opinion, to be free is to follow your own desires	0.20	-0.09	0.02	0.16	**0.38**	-0.02
33	Someone who is independent does not need government assistance to get by	0.13	0.21	0.27	-0.20	0.11	-0.02
34	To find a job, you have to accept relocation	0.14	0.24	0.09	-0.01	0.04	0.09
35	It is important that our happiness does not depend on others	0.07	0.02	0.02	0.32	**0.41**	-0.16
36	I think that competition is inevitable	-0.10	**0.70**	-0.04	0.13	0.01	0.04

37	Competition is what allows society to be efficient	0.04	**0.68**	0.13	0.00	-0.01	0.02
38	Competition is the best way to spot talented people	-0.04	**0.79**	0.01	-0.03	0.05	0.01
39	We need more competitiveness in society	0.04	**0.52**	0.20	-0.10	0.01	0.02
40	The competition is more a matter of one's mindset than of external pressures	0.25	0.20	0.06	-0.06	0.17	-0.04
41	Competition is the best way to encourage us to do our best	0.02	**0.73**	0.00	0.01	0.06	-0.04
42	Lowering taxes for the wealthiest allows them to invest and therefore to create wealth for all	0.13	0.15	**0.43**	-0.06	-0.08	0.06
43	A public service like Pôle Emploi* should be managed by a private company rather than by the State	-0.05	-0.01	**0.56**	0.08	0.02	0.09
44	The budget balance of France must be the main concern for the successive governments	0.14	0.06	0.29	0.04	0.23	-0.11
45	The State must let business owners manage their companies as they wish	0.06	0.08	**0.44**	-0.03	0.08	0.00
46	The fact that salary differences be based on individual merit seems justified to me	0.18	**0.32**	0.16	0.05	-0.09	-0.14
47	If one person possesses more than another, I assume that they deserve it	0.23	0.22	0.26	0.02	-0.03	0.01
48	The important thing is not that everyone has the same resources, but rather that everyone is satisfied with what they have	**0.37**	0.07	0.13	0.03	0.00	-0.11
49	If people are not doing well financially, it is most often because they do not know how to handle their money	0.28	0.08	0.34	-0.12	0.06	0.05
50	When people hold us back from our goals, it's best to let them go	-0.09	-0.09	0.20	**0.56**	0.28	0.17
51	It is better to part with people who waste our time	-0.14	-0.05	0.19	**0.52**	0.24	0.16
52	It's normal to socialize with certain people for the sole purpose of building a professional network	0.03	0.21	0.10	0.29	0.00	0.27
53	We should make more choices that really correspond to us without being influenced by others	0.13	-0.08	-0.04	**0.47**	0.21	-0.11
54	To be independent is to have no obligation toward anyone else	0.07	0.10	-0.02	-0.09	**0.62**	0.05
55	To be free is to have nothing to ask of anyone	0.05	0.09	-0.04	-0.09	**0.69**	0.05
56	One should not give up personal wishes for other people	0.10	-0.12	0.15	0.24	**0.37**	-0.02
57	You should never rest on your laurels, but always seek to improve	0.15	0.07	-0.02	**0.64**	-0.08	-0.13
58	It is more important to always strive for improvement than to be satisfied with a single success.	0.18	0.13	0.06	**0.55**	-0.10	-0.17

59	What gives meaning to one's life is to have clear personal goals.	0.31	0.07	0.06	**0.43**	0.00	-0.06
60	Multiplying new experiences is more important than keeping your habits	0.18	0.11	-0.06	**0.48**	-0.06	0.24
61	Ensuring that everyone has the same economic resources is not the responsibility of the State	0.18	0.22	**0.41**	0.00	-0.16	0.07
62	Higher education must be used to select the best and brightest for the job market	0.13	0.34	0.27	-0.05	-0.05	0.09
63	The State must guarantee the freedom of citizens rather than equality between citizens	0.04	0.18	**0.36**	-0.03	0.12	0.07
64	The State should spend less money on public services	-0.03	0.05	**0.68**	-0.03	-0.01	0.07
65	Reducing France's debt must be a top priority	0.08	0.09	**0.45**	0.05	0.13	-0.18
66	People who invest their money in a company should be rewarded more than those who work there	-0.02	0.28	0.32	-0.11	-0.03	0.25
67	Helping people in difficulty is the job of non-profit organizations, not the government	0.09	0.10	**0.56**	-0.12	-0.01	0.15
68	Privatizing public services would make them more efficient	-0.02	0.04	**0.69**	0.15	-0.03	-0.09
		Pct. of variance explained					
		8%	7%	7%	6%	5%	3%

5-factor structure

		EFA results				
		Factor loadings				
	**NOQ items**	**1**	**2**	**3**	**4**	**5**
1	It is only because we lack courage or self-confidence that we do not seize the opportunities offered to us	0.07	**0.45**	0.14	-0.11	0.00
2	With proper planning, all our goals are achievable	0.11	**0.55**	-0.01	0.01	0.04
3	The secret of success is to know yourself well	-0.11	**0.44**	0.12	0.19	0.05
4	When you encounter difficulties, the first thing to do is to question yourself	0.03	**0.35**	0.05	0.22	-0.07

5	It is mainly by working on ourselves that we can change the circumstances of our lives	0.03	**0.57**	0.09	0.01	0.06
6	With the right kind of motivation you can do anything	0.03	**0.67**	-0.04	0.13	-0.06
7	If we don't succeed, it's because we make bad choices	0.32	0.25	0.00	-0.14	0.11
9	Distinguishing myself from others through my personality is a priority to me.	0.06	0.14	0.23	0.07	**0.36**
10	To succeed, you have to know how to sort out the people around you	0.03	0.04	**0.51**	0.01	0.13
11	When a relationship does not benefit me, I prefer to put an end to it	-0.04	-0.07	**0.47**	0.18	0.12
13	It is important to regularly change our groups of friends in order to enrich ourselves on a personal level	0.21	-0.02	0.11	-0.14	**0.38**
14	I would be more successful in my endeavors without my close ones	0.24	-0.03	0.25	-0.27	0.29
15	To fulfill our personal wishes, we shouldn't get too attached to other people	0.17	0.11	**0.38**	-0.21	0.23
16	Continuous improvement is a priority for me	-0.06	0.21	0.04	**0.53**	0.19
17	To give meaning to my life I constantly set new goals	0.06	0.24	0.07	**0.36**	0.23
18	It is important to take risks in life	0.04	0.22	0.18	0.19	0.19
19	There is always something positive to be gained from any situation, even the worst	-0.10	**0.44**	-0.02	0.23	0.03
20	It is up to each person to adapt to all situations	0.19	**0.44**	0.05	0.00	-0.03
21	We can find solutions to all of the obstacles we encounter in life	0.09	**0.49**	0.03	0.07	-0.07
22	Rather than trying to change society, everyone should work on themselves	0.15	**0.38**	0.07	0.12	-0.06
23	To be happy, we simply need to focus on the positive	-0.07	**0.51**	0.05	-0.09	0.11
24	When we are not going well, we only need to change our perspective on the situation to feel better	0.07	**0.50**	-0.08	-0.04	0.11
26	Controlling our emotions in all circumstances is crucial	0.11	0.19	0.25	0.09	0.11
28	Depending on others makes us vulnerable	0.14	-0.03	**0.47**	0.12	-0.02
29	It is important not to depend on other people	0.01	0.08	**0.49**	0.16	-0.11
30	In my opinion, freedom is not having any obligations	-0.02	0.19	**0.42**	-0.31	0.05

31	You are truly free when you can control your emotions	0.11	0.22	0.23	-0.03	0.25
32	In my opinion, to be free is to follow your own desires	-0.08	0.24	**0.42**	0.00	-0.07
33	Someone who is independent does not need government assistance to get by	**0.43**	0.14	0.00	-0.17	-0.05
34	To find a job, you have to accept relocation	0.29	0.15	0.03	0.00	0.11
35	It is important that our happiness does not depend on others	-0.04	0.12	**0.49**	0.18	-0.13
36	I think that competition is inevitable	**0.50**	-0.04	0.00	0.23	0.22
37	Competition is what allows society to be efficient	**0.65**	0.07	-0.07	0.13	0.16
38	Competition is the best way to spot talented people	**0.61**	0.02	-0.05	0.12	0.19
39	We need more competitiveness in society	**0.60**	0.06	-0.07	0.01	0.09
40	The competition is more a matter of one's mindset than of external pressures	0.21	0.28	0.08	-0.04	-0.01
41	Competition is the best way to encourage us to do our best	**0.55**	0.08	-0.03	0.15	0.15
42	Lowering taxes for the wealthiest allows them to invest and therefore to create wealth for all	**0.54**	0.08	-0.05	-0.05	-0.03
43	A public service like Pôle Emploi* should be managed by a private company rather than by the State	**0.50**	-0.10	0.18	-0.05	-0.08
44	The budget balance of France must be the main concern for the successive governments	0.29	0.15	0.23	-0.02	-0.18
45	The State must let business owners manage their companies as they wish	**0.48**	0.04	0.12	-0.08	-0.11
46	The fact that salary differences be based on individual merit seems justified to me	**0.40**	0.17	-0.13	0.19	-0.06
47	If one person possesses more than another, I assume that they deserve it	**0.43**	0.21	-0.02	0.06	0.00
48	The important thing is not that everyone has the same resources, but rather that everyone is satisfied with what they have	0.17	**0.36**	-0.04	0.09	-0.09
49	If people are not doing well financially, it is most often because they do not know how to handle their money	**0.40**	0.26	0.03	-0.13	-0.04
50	When people hold us back from our goals, it's best to let them go	0.07	-0.10	**0.63**	0.23	0.06
51	It is better to part with people who waste our time	0.09	-0.16	**0.58**	0.23	0.07
52	It's normal to socialize with certain people for the sole purpose of building a professional network	0.27	0.01	0.20	0.17	0.26

53	We should make more choices that really correspond to us without being influenced by others	-0.16	0.14	**0.38**	0.34	-0.09
54	To be independent is to have no obligation toward anyone else	0.03	0.15	**0.52**	-0.26	0.02
55	To be free is to have nothing to ask of anyone	0.01	0.14	**0.58**	-0.29	0.02
56	One should not give up personal wishes for other people	-0.01	0.11	**0.49**	0.03	-0.11
57	You should never rest on your laurels, but always seek to improve	-0.01	0.12	0.18	**0.62**	-0.04
58	It is more important to always strive for improvement than to be satisfied with a single success.	0.11	0.16	0.10	**0.58**	-0.08
59	What gives meaning to one's life is to have clear personal goals.	0.08	0.29	0.16	**0.40**	-0.02
60	Multiplying new experiences is more important than keeping your habits	0.04	0.15	0.20	**0.37**	0.26
61	Ensuring that everyone has the same economic resources is not the responsibility of the State	**0.58**	0.12	-0.11	0.05	0.02
62	Higher education must be used to select the best and brightest for the job market	**0.55**	0.12	-0.06	0.00	0.09
63	The State must guarantee the freedom of citizens rather than equality between citizens	**0.48**	0.03	0.15	-0.09	-0.01
64	The State should spend less money on public services	**0.67**	-0.09	0.11	-0.12	-0.12
65	Reducing France's debt must be a top priority	**0.47**	0.06	0.15	0.04	-0.27
66	People who invest their money in a company should be rewarded more than those who work there	**0.56**	-0.05	0.01	-0.14	0.18
67	Helping people in difficulty is the job of non-profit organizations, not the government	**0.62**	0.04	0.04	-0.18	-0.02
68	Privatizing public services would make them more efficient	**0.65**	-0.09	0.13	0.09	-0.24
		Pct. of variance explained				
		11%	8%	7%	5%	2%
